# Chromatin Profiles of Chromosomally Integrated Human Herpesvirus-6A

**DOI:** 10.3389/fmicb.2019.01408

**Published:** 2019-06-26

**Authors:** Anthony J. Saviola, Cosima Zimmermann, Michael P. Mariani, Sylvia A. Signorelli, Diana L. Gerrard, Joseph R. Boyd, Darren J. Wight, Guillaume Morissette, Annie Gravel, Isabelle Dubuc, Louis Flamand, Benedikt B. Kaufer, Seth Frietze

**Affiliations:** ^1^Department of Biomedical and Health Sciences, University of Vermont, Burlington, VT, United States; ^2^Institute of Virology, Department of Veterinary Medicine, Freie Universität Berlin, Berlin, Germany; ^3^Department of Biochemistry and University of Vermont Cancer Center, University of Vermont College of Medicine, Burlington, VT, United States; ^4^Department of Microbiology, Infectious Disease and Immunology, Université Laval and CHU de Quebec Research Center-Université Laval, Quebec, QC, Canada

**Keywords:** ChIP-seq, HHV-6A, iciHHV-6A, latency, MNase-seq, nucleosomes, RNA-seq

## Abstract

Human herpesvirus-6A (HHV-6A) and 6B (HHV-6B) are two closely related betaherpesviruses that are associated with various diseases including seizures and encephalitis. The HHV-6A/B genomes have been shown to be present in an integrated state in the telomeres of latently infected cells. In addition, integration of HHV-6A/B in germ cells has resulted in individuals harboring this inherited chromosomally integrated HHV-6A/B (iciHHV-6) in every cell of their body. Until now, the viral transcriptome and the epigenetic modifications that contribute to the silencing of the integrated virus genome remain elusive. In the current study, we used a patient-derived iciHHV-6A cell line to assess the global viral gene expression profile by RNA-seq, and the chromatin profiles by MNase-seq and ChIP-seq analyses. In addition, we investigated an *in vitro* generated cell line (293-HHV-6A) that expresses GFP upon the addition of agents commonly used to induce herpesvirus reactivation such as TPA. No viral gene expression including miRNAs was detected from the HHV-6A genomes, indicating that the integrated virus is transcriptionally silent. Intriguingly, upon stimulation of the 293-HHV-6A cell line with TPA, only foreign promoters in the virus genome were activated, while all HHV-6A promoters remained completely silenced. The transcriptional silencing of latent HHV-6A was further supported by MNase-seq results, which demonstrate that the latent viral genome resides in a highly condensed nucleosome-associated state. We further explored the enrichment profiles of histone modifications *via* ChIP-seq analysis. Our results indicated that the HHV-6 genome is modestly enriched with the repressive histone marks H3K9me3/H3K27me3 and does not possess the active histone modifications H3K27ac/H3K4me3. Overall, these results indicate that HHV-6 genomes reside in a condensed chromatin state, providing insight into the epigenetic mechanisms associated with the silencing of the integrated HHV-6A genome.

## Introduction

Human herpesvirus 6 (HHV-6) was first discovered in patients with lymphoproliferative disorders (Salahuddin et al., 1986) and has a seroprevalence of more than 90% (Zerr et al., 2005). Since then, two variants were identified termed HHV-6A and HHV-6B, which have been classified as two distinct species based on their biological, immunological, and molecular properties (Adams and Carstens, 2012; Ablashi et al., 2014). Infection with HHV-6B occurs within the first 2 years of life and is the primary cause of *Roseola infantum*, a febrile illness with a skin rash that can be accompanied by seizures, meningoencephalitis, and encephalopathy (Yamanishi et al., 1988; De Bolle et al., 2005). The pathologies associated with HHV-6A remain poorly characterized, although a recent report has suggested an association between HHV-6A and Alzheimer’s disease (Readhead et al., 2018).

Upon primary infection, HHV-6A/B establishes a lifelong persistent infection in the host, termed latency (Takahashi et al., 1989; Lusso et al., 1991). In latently infected cells, both viruses integrate their genome into the telomere region of host chromosomes (Arbuckle et al., 2010, 2013). This integration is facilitated by telomeric repeats (TTAGGG)_n_ at the ends of the virus genome (Kaufer et al., 2011; Kaufer and Flamand, 2014; Wallaschek et al., 2016); however, the viral and/or cellular proteins that mediate integration remain elusive. Aside from latently infected cells, both viruses can also integrate their genomes into germ cells. This allows vertical transmission of HHV-6A/B and consequently individuals harbor the integrated virus in every cell of their body (Pellett et al., 2012). Approximately 1% of the human population has this condition termed inherited chromosomally integrated HHV-6A/B (iciHHV-6) (Pellett et al., 2012). The clinical consequences for iciHHV-6 patients remain poorly understood. An analysis of a large cohort revealed that iciHHV-6 patients have an increased risk of developing angina pectoris and other diseases (Gravel et al., 2015), but more research is needed to provide a better understanding of these disease associations. Reactivation of HHV-6A/B is associated with a number of diseases including encephalitis, multiple sclerosis, and graft rejection following transplantation (De Bolle et al., 2005; Caselli and Di Luca, 2007). For example, reactivation occurs in 30–70% of hematopoietic stem cell transplantation (HSCT) recipients and is linked to graft rejections and higher mortality (Vinnard et al., 2009; Hill and Zerr, 2014; Winestone et al., 2018). In addition, a higher frequency and severity of both graft-versus-host disease (GvHD) and cytomegalovirus (CMV) viremia were observed in HSCT patients when either the recipient or donor were iciHHV-6 positive (Hill et al., 2017).

Analysis of iciHHV-6 cell lines by RT-qPCR revealed that few or none of the assessed genes are expressed from the integrated virus genome (Strenger et al., 2014), suggesting that the integrated genome is efficiently silenced. Epigenetic modifications likely contribute to this silencing and will be the focus of this manuscript. Chromatin dynamics are largely mediated by a variety of post-translational modifications of the N-terminal tail of histone proteins, which promotes either an active or repressive transcriptional state. Modifications such as trimethylation (me3) of lysine 4 (K4) of histone 3 (H3K4me3), and acetylation (ac) of lysine 27 (K27) of histone 3 (H3K27ac), are often associated with less condensed chromatin referred to as the transcriptionally active euchromatin. On the other hand, modifications such as H3K27me3 and H3K9me3 are often associated with highly condensed chromatin and a repressive transcriptional state referred to as heterochromatin (Berger, 2007; Li et al., 2007). Heterochromatin formation and nucleosomal occupancy have been linked to viral latency for herpes simplex virus 1 (HSV-1) (Deshmane and Fraser, 1989; Kubat et al., 2004; Wang et al., 2005; Knipe and Cliffe, 2008; Cliffe et al., 2009; Bloom et al., 2010), Epstein-Barr virus (Dyson and Farrell, 1985; Shaw, 1985; Moquin et al., 2018), and human immunodeficiency virus (HIV) (Jordan et al., 2003; du Chéné et al., 2007; Friedman et al., 2011). However, the epigenetic modifications that contribute to the silencing of integrated (latent) HHV-6A/B remain unknown.

In the current study, we employed unbiased genomic approaches using RNA-, MNase-, and ChIP-sequencing to explore the state of the viral genome in patient-derived iciHHV-6 and experimentally infected 293-HHV-6A cells. Our data reveal that integrated HHV-6A is entirely transcriptionally silent and exists in a highly condensed, nucleosome-associated state. Further, the repressive histone modifications H3K9me3 and H3K27me3 were detected across the HHV-6A genome, although both histone modifications were not significantly enriched. Additionally, the latent genome lacks the active histone modifications H3K27ac and H3K4me3. These results provide the first chromatin landscape of the integrated HHV-6A genome in experimentally infected and iciHHV-6 patient-derived cells.

## Materials and Methods

### Cell Lines and Virus

Following approval by the CHU de Quebec-Université Laval ethics review board and patients consent, umbilical cords of women undergoing C-section were tested for the presence of iciHHV-6A as described previously (Gravel et al., 2015). Smooth muscle cells (SMCs) were obtained from the arteries of iciHHV-6A+ umbilical cords as described (Moreau et al., 2007) and immortalized by transduction with a lentiviral vector expressing SV40 T antigens (Addene #22298). Human SMCs (iciHHV-6A) were cultured in Dulbecco’s modified Eagle’s medium (DMEM) supplemented with 20% fetal bovine serum (FBS), 1% penicillin–streptomycin (Pen/Strep), and 1% L-Glutamine. Human epithelial kidney 293 T (293 T, ATCC CRL-11268) cells were cultured in the same medium but supplemented with 10% FBS. All cells were maintained in 10 cm^2^ flasks as a monolayer culture in a humidified 5% CO_2_ air incubator at 37°C. Bacterial artificial chromosome (BAC)-derived HHV-6A (strain U1102) expressing green fluorescent protein (GFP) under the control of the HCMV major immediate early (IE) promoter (HHV-6-GFP) was propagated in JJHan cells as described previously (Tang et al., 2010). 293 T cells were infected with HHV-6-GFP and GFP positive cells were isolated using a FACS AriaIII cell sorter (BD Biosciences). Clones harboring the integrated HHV-6A genome (293-HHV-6A) were identified by quantitative PCR (qPCR) and confirmed by fluorescent *in situ* hybridization (FISH). To investigate whether expression of genes from the integrated virus can be induced, clonal 293-HHV-6A cells were treated with either phorbol 12-myristate 13 acetate (TPA, Sigma) (10 ng/ml), Trichostatin A (TSA, Sigma) (0.25 μM), sodium butyrate (NaBy, Sigma) (3 mM), Etoposide (ETP, Sigma) (0.5 μM), suberoylanilide hydroxamic acid (SAHA; also known as vorinostat, Sigma) (1 μM), Forskolin (FSK, Sigma) (10 μM), or hydrocortisone (Dexamethasone, Dexa) (10 μM) for 24 h. Reactivation of HHV-6A was monitored using FACS Calibur (BD Biosciences) to determine the percent of GFP positive (GFP+) cells.

### Fluorescent *in situ* Hybridization

To prove that HHV-6A genome is present at the ends of metaphase chromosomes, FISH was performed as described previously (Rens et al., 2006; Kaufer et al., 2011; Kaufer, 2013). Briefly, cell cultures were treated with 0.05 μg/ml colcemid (Gibco) overnight to arrest the cells in metaphase. Cells were collected by centrifugation, resuspended in hypotonic solution (0.075 M KCl) followed by methanol/acetic acid fixation and stored at −20°C until further use. Metaphase spreads were generated as described previously (Kaufer et al., 2011). The virus genome was detected using a HHV-6A-specific digoxigenin-labeled probe and detected using different antibodies as described by Wight et al. (2018). Slides were mounted using DAPI Vectashield (Vector Laboratories) and images taken with an Axio Imager M1 (Zeiss).

### Quantitative PCR

HHV-6 genome copies were determined by qPCR using specific primers and TaqMan probes for U94 as described previously (Wallaschek et al., 2016). Briefly, DNA was isolated using the RTP® DNA/RNA Virus Mini Kit (Stratec) according to manufacturer’s instructions. U94 gene copy numbers were normalized against the genome copies of the cellular ß_2_M gene and compared to a control cell line (AP3) harboring one copy of the HHV-6A genome per cell (Gravel et al., 2017).

### RT-qPCR

For HHV-6A transcriptome quantification, cells were treated with TPA during 6 days and RNA was isolated using the RNeasy Plus Mini Kit (Qiagen) according to manufacturer’s instructions. Total RNA was reverse transcribed to single-stranded cDNA using High-Capacity cDNA Reverse Transcription Kit (Applied Biosystems) according to manufacturer’s instructions. qPCR was performed using TaqMan probes for immediate-early (U86, U90) and early genes (U41, U70) as described previously (Wight et al., 2018). Gene copy numbers were normalized against the genome copies of the cellular ß_2_M gene.

### RNA-seq

RNA was extracted from patient-derived iciHHV-6 cells using Trizol Reagent (Life Technologies) and purified using Direct-zol RNA MicroPrep Kit (Zymo Research #R2060) following the manufacturer’s instructions. In addition, RNA was isolated and purified from *in vitro* generated HHV6-GFP cells treated with either DMSO or 10 ng/ml phorbol 12-myristate 13 acetate (TPA, Sigma) for 24 h at 37°C in duplicate. One microgram of total RNA was depleted of ribosomal RNA (rRNA) using the KAPA RiboErase Kit (#KR1142) and libraries were prepared using the KAPA Stranded RNA-seq Library Preparation Kit (#KR0934). Libraries were quantified using Qubit (Life Technologies), and quality was assessed using the Agilent Bioanalyzer High-Sensitivity DNA kit (Agilent Technologies). Barcoded libraries were pooled and sequenced on an Illumina HiSeq 2,500 at the Genomics Core Facility (University of Texas Health Science Center, San Antonio, TX) to obtain 50-bp single-end reads. RNA-seq data were processed using TopHat as described previously (Trapnell et al., 2009) using human (hg38), HHV-6A (NC_001664.2) and the custom HHV-6A-GFP BAC genome. BAM files were sorted and converted to SAM files using SAMtools (Li et al., 2009) and reads were counted with HTSeq against the corresponding Gencode GTF files (Anders et al., 2015). Differential analysis of RNA-seq count data between TPA- and DMSO-treated cells was performed using DESeq2 (Love et al., 2014) with a gene false discovery rate of <0.5% (FDR < 0.005) and a fold change >2 considered as significantly different. RNA-seq datasets were visualized using the Integrated Genome Browser (Nicol et al., 2009).

### MicroRNA-seq

RNA was extracted and purified from iciHHV-6A, and TPA or DMSO treated 293-HHV-6A cells as described above. MicroRNA (miRNA) libraries were prepared using NEBNext Multiplex Small RNA Sample Prep for Illumina (#E7300) following the manufacturer’s protocol. Library quantification and quality were assessed as described above, and pooled libraries were sequenced on an Illumina HiSeq 2,500 at the Genomics Core Facility (University of Texas Health Science Center, San Antonio, TX) to obtain 50-bp single-end reads. miRNA data were processed using the Oasis2 package (Rahman et al., 2018).

### Micrococcal Nuclease-seq

Micrococcal nuclease (MNase)-seq was performed using the EZ Nucleosomal DNA Prep Kit (Zymo Research #D5220) following the manufacturer’s protocol. Briefly, nuclei isolated from 1 million iciHHV-6A and 293-HHV-6A cells were treated with varying concentrations of MNase (final concentrations of 0.07, 0.1, 0.2, 0.3 U/100 μl reaction) and incubated at room temperature for 5 min. The reaction was terminated with MNase stop buffer, and following nucleosomal DNA purification, libraries for each individual titration were prepared with the NEB Ultra II Library Prep Kit (#E7645S) following the manufacturer’s protocol. Libraries were purified using 0.8X AMPure beads and quantified using Qubit (Life Technologies). Library quality and presence of mono-, di-, and tri-nucloesomes were observed using the Agilent 2,100 Bioanalyzer High-Sensitivity DNA kit. Barcoded libraries were pooled and sequenced on an Illumina HiSeq 2,500 instrument lane at the Genomics Core Facility (University of Texas Health Science Center, San Antonio, TX) to obtain 80-bp paired-end reads. Sequences were aligned to the human (hg38), HHV-6A (NC_001664.2), and custom HHV-6A-GFP genome using Bowtie2 (Langmead and Salzberg, 2012), and the bamCoverage command in deepTools was used to filter out reads with insert sizes <50 bp and >500 bp and to generate bigwigs (Ramírez et al., 2016). Profiles were generated for individual titration points, and as it has been previously reported that nucleosome occupancy can change across the genome in response to MNase treatment (Maehara and Ohkawa, 2016; Mieczkowski et al., 2016; Mueller et al., 2017), data for each titration were merged based on the cell line. Data were processed using DANPOS as described previously (Chen et al., 2013).

### Chromatin Immunoprecipitation

The antibodies used for ChIP-seq experiments were: histone H3 [#2650 (Cell Signaling Technologies)], H3K4me3 [#8580 (Abcam, lot GR273043)], H3K27ac [#4729 (Abcam, lot GR288020)], H3K27me3 [#6002 (Abcam, lot GR275911-4)], H3K9me3 [#8898 (Abcam, lot GR21638-1)], and control IgG [#171870 (Abcam)]. ChIP sequencing was performed as described previously (O’Geen et al., 2010) on the iciHHV-6A cell lines. For all antibodies, 20 μg of chromatin was incubated with 4 μl of antibody overnight at 4°C. The complexes were precipitated with 20 μl of Pierce™ Protein A/G Magnetic Beads, followed by extensive washing, and final elution of the immunoprecipitated chromatin complexes in 100 μl of ChIP elution buffer for subsequent DNA purification and library construction. Libraries were prepared using the NEBNext ChIP-Seq Library Prep Master Mix Set for Illumina (#E6240) following the manufacturer’s instructions. Library concentration and quality were assessed on a Qubit (Life Technologies) and an Agilent 2,100 Bioanalyzer, respectively. Pooled libraries were sequenced on an Illumina HiSeq 2,500 instrument lane at the Genomics Core Facility (University of Texas Health Science Center, San Antonio, TX) to obtain 50-bp single-end reads. Sequence reads were aligned to the human (hg38) and HHV-6A (NC_001664.2) genomes using Bowtie2 (Langmead and Salzberg, 2012). The resulting SAM alignment files were used for peak calling using MACS2 against the control (no antibody) inputs (Zhang et al., 2008) and using default parameters. ChIP DNA assayed by quantitative PCR is expressed as fold enrichment over an internal control (GAPDH) and is normalized to input. Primers for ChIP-qPCR are listed in Table 3.

## Results

### Gene Expression Profiling of Integrated HHV-6A Genomes

**Figure 1:**
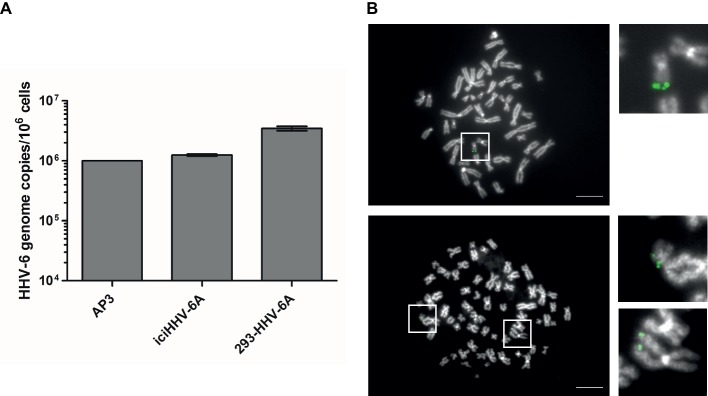
Detection of HHV-6A genome at the end of methaphase chromosomes of iciHHV-6 and 293-HHV-6A cell lines. **(A)** HHV-6A (U94) genome copy numbers were detected by qPCR. Copy numbers per million cells are shown as means of three independent experiments with standard deviation. AP3 (HHV-6A) cells were used as a control. Copy numbers were normalized to AP3 cells carrying one HHV-6A copy per cell. **(B)** HHV-6A genome visualized by FISH. Representative metaphase images of iciHHV-6A (upper panel) and 293-HHV-6A (lower panel) cell lines. The HHV-6A genome was detected using a specific DIG-labeled probe (green) and chromosomes were visualized using DAPI (gray). Scale bar corresponds to 10 μm.

To determine the gene expression of integrated HHV-6A genomes, we employed a global RNA-seq approach using clonal iciHHV-6A patient cells (iciHHV-6A) and an *in vitro* generated cell line (293-HHV-6A). The latter cell model was obtained by infecting 293 T cells with HHV-6A expressing GFP under the control of the major immediate-early (IE) HCMV promoter. Presence of the HHV-6A genome in both cell lines was confirmed by qPCR and FISH analyses (Figures 1A,B). To assess gene expression, we collected total RNA from latent cell cultures, constructed stranded rRNA-depleted Illumina RNA-seq libraries and mapped the reads to the human and the HHV-6A reference genomes. Characteristic of a typical RNA-seq profile, we detected a range of host cellular transcripts with RPKM (reads per kb per million mapped) values ranging from 1 to >1,000 RPKM in both iciHHV-6A (Supplementary Figure S1A) and 293-HHV-6A cell lines (Supplementary Figure S1B, DMSO treatment). Surprisingly, when our data were mapped to the HHV-6A reference genomes, we failed to detect any previously determined viral latency-associated transcripts in both HHV-6A cell lines with all RPKM values <1.0. To confirm that the absence of transcripts was not due to major changes in the virus genome, we performed whole genome sequencing and genome-guided assembly. No deletion or mutations were observed that would explain the complete absence of viral transcripts. These results demonstrate a tight silencing of the entire HHV-6A genome in both patient-derived and an *in vitro* generated cell line.

**Figure 2:**
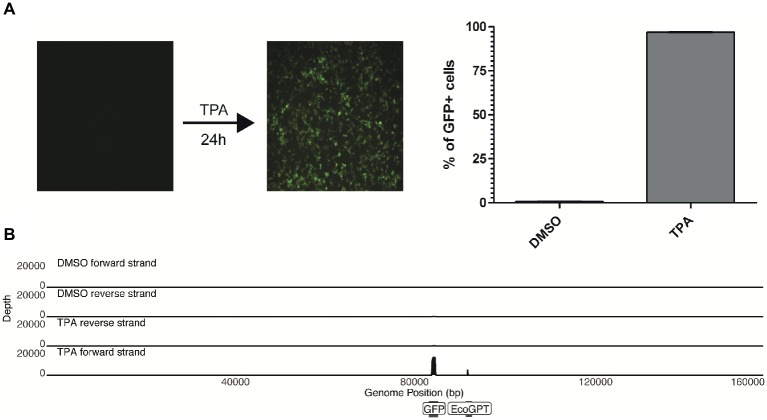
TPA stimulation fails to reactivate *in vitro* chromosomally integrated HHV-6A. **(A)** 293-HHV-6A cells were treated with TPA or DMSO for 24 h. GFP expression was measured by flow cytometry. Mean of the % of GFP+ cells from two independent experiments is shown. **(B)** RNA was extracted from DMSO- or TPA-treated 293-HHV-6A cells and strand-specific RNA-seq libraries were generated. Alignment to the HHV-6A-GFP genome revealed that only transcripts associated with the GFP and the EcoGPT regions of the recombinant genome are detectable following TPA stimulation.

Upon treatment of the 293-HHV-6A cell line with common reagents that induce reactivation (Supplementary Figure S2), greater than 90% of the TPA-treated cells express GFP while fewer than 1% express GFP in the DMSO-treated control (Figure 2A). Therefore, we set to determine how TPA treatment affects the global expression profile of the virus genome in 293-HHV-6A cells as described above. As expected, TPA induced global changes on human gene expression compared to the control (Supplementary Figures S1B–D). However, only the foreign HCMV IE and HSV-1 TK promoters driving GFP and EcoGPT respectively were induced in the HHV-6A genome, while all HHV-6A promoters remained silent (Figure 2B). To confirm this observation, we assessed the expression of selected viral genes following TPA induction for an extended period of time by RT-qPCR (Supplementary Figure S2B). Our data show that foreign but not HHV-6A promoters are efficiently induced by TPA, suggesting a selective activation by this activation stimulus.

Since viral encoded miRNAs play a pivotal role in the viral life cycle and latency of other herpesviruses, we also investigated the miRNA expression of these cells by small RNA-seq analysis. We performed small RNA-seq analysis using the patient-derived and experimental infected models (treated with TPA or DMSO control). Following adapter removal, we obtained a high number of reads that uniquely align to the human reference genome, the majority of these reads being identified as miRNAs (Supplementary Figure S3). No HHV-6A-derived miRNAs were identified in any of the libraries, indicating that HHV-6A does not express miRNAs in the cell lines we have tested. Taken together, our results suggest that both *in vitro* generated and patient-derived iciHHV-6A cells are transcriptionally silent. Moreover, this demonstrates that TPA stimulation fails to efficiently activate the HHV-6A promoters while foreign promoters in the viral genome are activated.

### Chromosomally Integrated HHV-6A Is Associated With Nucleosomes

To better understand the transcriptional silencing observed from integrated HHV-6A, we examined the association of the virus genome with nucleosomes. Nucleosome positioning and occupancy profoundly influence gene expression (Li et al., 2007; Jiang and Pugh, 2009). Therefore, mapping the genomic location of nucleosomes is critical for understanding the mechanisms of chromatin-mediated transcriptional regulation. A commonly used approach is MNase-seq, in which nucleosome-free regions of chromatin are digested with MNase leaving nucleosome-associated DNA intact (Cui and Zhao, 2012; Mieczkowski et al., 2016; Pajoro et al., 2018). Mapping these data to a reference genome allows for a genome-wide profiling of nucleosome occupancy.

**Figure 3:**
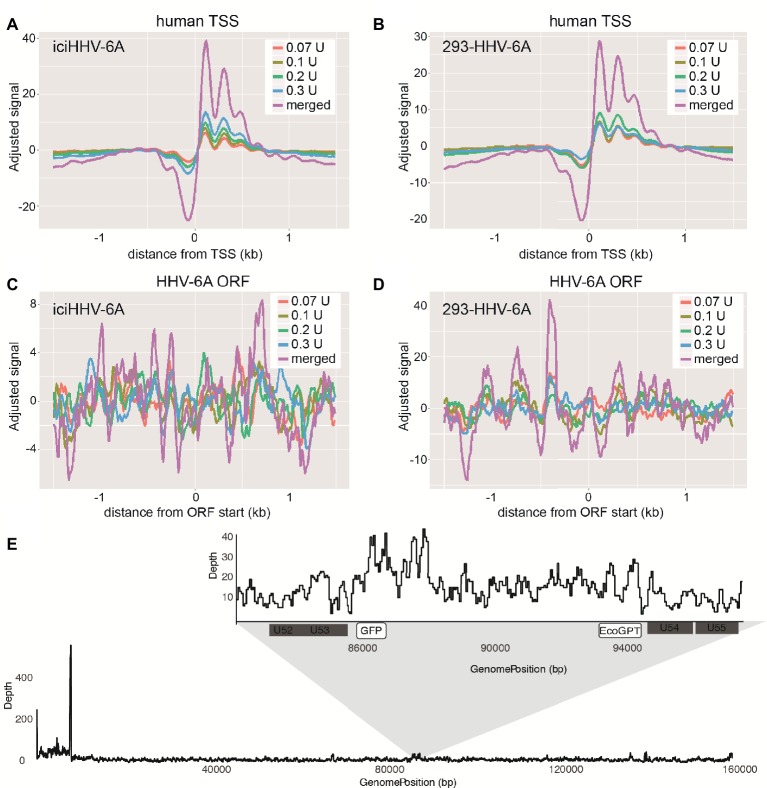
Latent chromosomally integrated HHV-6A is associated with nucleosomes. The genome-wide distribution of the average nucleosomal occupancy in patient-derived iciHHV-6A and *in vitro* infected 293-HHV-6A cell lines. **(A,B)** Alignment to all transcription start sites (TSSs) for the human genome (hg38). For both cell lines, there is decreased nucleosome signal at all active TSSs across the hg38 genome for the four MNase titrations (0.07, 0.1, 0.2, 0.3 U), and for the merged datasets, validating our MNase-seq experiments. **(C,D)** Alignment to all open reading frame (ORF) start sites of the **(C)** iciHHV-6A (NC_001664.2) and 293-HHV-6A **(D)** genomes, respectively. Both cell lines demonstrate enriched nucleosome signal downstream and upstream from all ORF start sites found throughout the HHV-6A genome. **(E)** A zoomed-in snapshot showing nucleosomal enrichment across the 293-HHV-6A GFP and EcoGPT regions.

To profile the nucleosomal landscape of latent HHV-6A, we performed MNase-seq on non-TPA-treated ciHHV-6 samples with four different MNase concentrations (0.07, 0.1, 0.2, and 0.3 U). MNase-seq reads were aligned to both the human (hg38) and HHV-6A reference genomes (Table 1) and we determined the distribution of nucleosome occupancy around the transcription start sites (TSSs), which are nucleosome-free regions of the genome (Buenrostro et al., 2013; Mieczkowski et al., 2016). As previously demonstrated, there is decreased nucleosome signal at all active TSSs across the human genome for each of the four MNase titrations, and for the merged datasets (Figures 3A,B; Maehara and Ohkawa, 2016; Mieczkowski et al., 2016). In contrast, the MNase-seq data revealed that the HHV-6A genome has limited MNase accessibility (Figures 3C,D), indicating that in both HHV-6A cell lines, the majority of the viral genome is in a highly compacted heterochromatin state. Further, the GFP and EcoGPT regions of the 293-HHV-6A cell lines also exhibit a nucleosome-dense, heterochromatic formation (Figure 3E), which correlates with the lack of observable GFP in the untreated cells (Figure 2A). Overall, our results suggest that all major regions reside in a highly condensed nucleosome-associated state that likely contributes to the transcriptionally silent state of the virus genome.

**Table 1:** MNase-seq alignment statistics for both iciHHV-6A and 293-HHV-6A cell lines.

iciHHV-6A	Total reads	Mapped to hg38	Mapped to HHV-6A
0.07 U	108,672,380	104,671,364	3,119
0.1 U	129,284,350	123,825,376	3,842
0.2 U	115,427,930	107,820,045	3,030
0.3 U	139,973,048	130,960,010	3,905
**293-HHV-6A**			
0.07 U	115,412,822	110,409,085	5,635
0.1 U	117,406,422	109,932,492	5,777
0.2 U	110,616,804	100,882,910	5,766
0.3 U	91,897,360	74,527,515	3,957

iciHHV-6A sequences were mapped to the HHV-6A (NC_001664.2) genome, whereas 293-HHV-6A sequences were aligned to a custom HHV-6A genome.

### Repressive Histone Modifications Are Detected Across the Latent iciHHV-6A Genome

**Figure 4:**
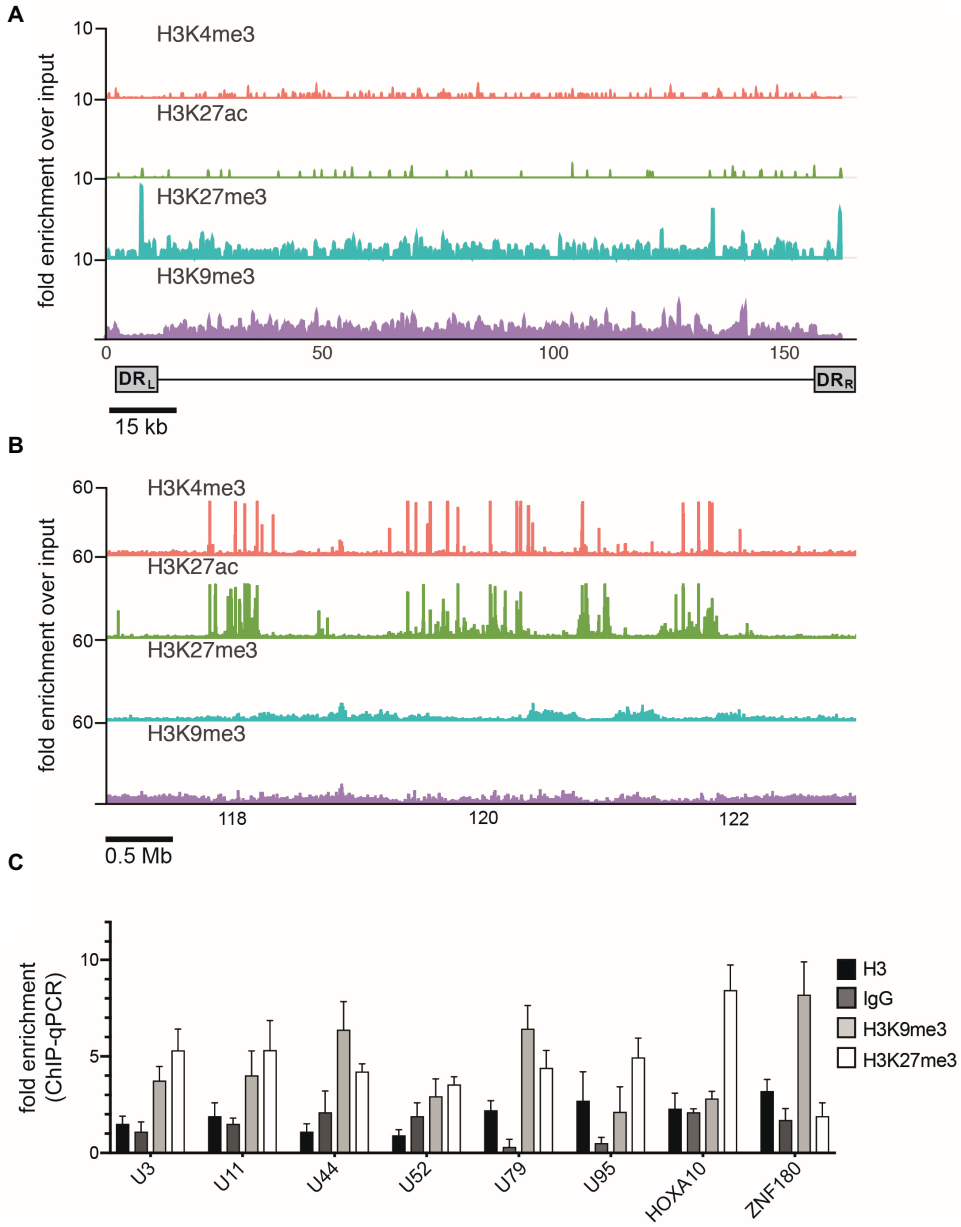
ChIP enrichment profiles of histone modifications across the iciHHV-6A and human genomes. **(A)** The ChIP-seq enrichment patterns of two active (H3K27ac and H3K4me3) and two repressive modifications (H3K9me3 and H3K27me3) aligned to the HHV-6A (NC_001664.2) genome. The scale represents the fold enrichment of ChIP over input. **(B)** The ChIP-seq enrichment patterns of histone modification ChIP-seq datasets aligned to the human (hg38) genome. The ChIP-seq binding patterns demonstrate a typical profile of broad enrichment patterns of H3K27me3 and H3K9me3 in regions devoid of the active histone marks H3K27ac and H3K4me3. The locus at the indicated region of chromosome 2 of the hg38 human genome in mega base pairs is shown (Mb). **(C)** ChIP-qPCR assays using primers targeting HHV-6 gene regions U3, U11, U44, U52, U79, U95 and the cellular gene regions HOXA10 and ZNF180. Chromatin was immunoprecipitated with nonspecific IgG, total histone H3, H3K9me3, or H3K27me3 antibodies. Data are shown as the fold-enrichment over GAPDH normalized by input from three independent ChIP assays (mean ± SD).

Mounting evidence indicates that regulatory histone modifications play central roles in viral latency and chromatin structure, including for both HSV-1 and HIV (Kubat et al., 2004; du Chéné et al., 2007). The density of nucleosome seeding of the integrated HHV-6A genome suggests that the virus may be associated with repressive histone modifications. Therefore, to identify potential epigenetic modifications that may regulate gene expression of the HHV-6A genome, we assayed the distribution of representative active (H3K27ac and H3K4me3) and repressive (H3K9me3 and H3K27me3) histone marks by a genome-wide ChIP-seq analysis in the patient-derived iciHHV-6 cell line. ChIP-seq reads were mapped to the HHV-6A genome and significantly enriched peaks were called against input controls. No enrichment of the active H3K27ac and H3K4me3 histone marks was detected across the HHV-6A genome (Figure 4A) as compared to the human genome (Figure 4B). In addition, the two repressive histone modifications H3K9me3 and H3K27me3 appeared to be present, but not significantly enriched compared to the input controls following peak calling using different peak calling algorithms with a range of parameters. This result may be due to the low number of overall reads mapped to the viral genome (Table 2). To further validate these findings, we performed ChIP-qPCR using primers specific to several HHV-6A genomic regions (Table 3). The repressive marks H3K9me3 or H3K27me3 are enriched over nonspecific IgG or pan-histone H3 antibodies, albeit at lower levels than cellular regions (Figure 4C). Taken together, these results suggest that the repressive histone modifications H3K9me3 and H3K27me3 are present on the latent virus genome and may be involved in chromatin-mediated repression of integrated HHV-6A gene expression.

**Table 2:** ChIP-seq alignment statistics for the iciHHV-6A cell line.

iciHHV-6A	Total reads	Mapped to hg38	Mapped to HHV-6A
H3K4me3	16,934,452	15,997,762	342
H3K27ac	26,765,754	24,673,031	2,379
H3K9me3	9,538,766	8,845,283	1,443
H3K27me3	22,923,694	22,382,904	492

Sequences were mapped to the HHV-6A (NC_001664.2) genome using Bowtie2.

**Table 3:** ChIP-qPCR primers.

Target	F primer	R primer
ZNF180	TGATGCACAATAAGTCGAGCA	TGCAGTCAATGTGGGAAGTC
GAPDH	CACCGTCAAGGCTGAGAACG	ATACCCAAGGGAGCCACACC
U3	ACAAACTGCAGCGATGACAC	TATGCGCACACGTGGTTATT
U11	TCCACACCGTTCGTATTCAA	AATCTGGATCTGCCGTTGTC
U44	GAGTTGGATCCGATTCTCCA	TTCAGACTCAACGCGTATCG
U52	GGGACACGGTTCAAAAAGAA	GCCCATGCTCTAAATCGAAA
U79	GCATATGGGTCATTTGACGA	TCACACGTTCCAGAGTCACC
U95	GTCGGATACAGACAGCGACA	TCTCTTGGCTTGGCGATACT

## Discussion

HHV-6A/B has been shown to integrate its genomes into the telomere region of latently infected cells, while most other human herpesviruses maintain their genomes as extrachromosomal circular episomes during latency (Grinde, 2013). Latent episomal herpesvirus genomes are usually epigenetically silenced during latency; however, the transcriptional activity and epigenetic state of the telomere-integrated HHV-6A/B genome remain poorly characterized. In the current study, we used unbiased genomic methodologies to analyze viral gene expression and the epigenetic state of the latent HHV-6A genome in patient-derived and *in vitro* infected cell lines. Our work provides several lines of evidence that the latent HHV-6A genome is maintained in a transcriptionally silent and highly compacted state, associated with the repressive histone modifications H3K9me3 and H3K27me3. Overall, these findings further advance our understanding of the chromatin-based mechanisms regulating HHV-6A gene expression and latency.

Although previous studies have linked low levels of viral transcripts to herpesvirus latency (Collins-McMillen and Goodrum, 2017), our RNA-seq analysis demonstrates that viral associated transcripts were not detected from the integrated HHV-6A genome in both iciHHV-6A and 293-HHV-6A cells. A previous study described four HHV-6B latency-associated transcripts (LATs) in latently infected cells (Kondo et al., 2002); however, these LATs were barely detectable in a more sensitive assay and showed maximal expression during the establishment of latency, and just before the onset of reactivation both *in vivo* and *in vitro* (Kondo et al., 2003). Therefore, the detection of HHV-6A LATs may be dependent on specific time points assayed, as we were unable to detect any HHV-6A-associated transcripts in both *in vitro* generated and iciHHV-6 patient cell lines. Furthermore, only the U94 gene was transcribed in freshly isolated peripheral blood mononuclear cells from HHV-6A-infected adults and *in vitro* infected cells, whereas other viral transcripts were not detected (Rotola et al., 1998). For alphaherpesviruses, HSV latency is associated with the expression of LATs in some latently infected neurons; however, LAT equivalent transcripts are not expressed in varicella zoster virus (VZV) latency (Kinchington et al., 2012), although a recently discovered latency transcript antisense of ORF61 has been reported (Depledge et al., 2018). Varying degrees of evidence on LATs in both natural HCMV infection and experimental latency models have been reported (Cheng et al., 2017; Shnayder et al., 2018), suggesting that herpesvirus latency may be more complex and dynamic than previously assumed (Goodrum, 2016; Collins-McMillen and Goodrum, 2017).

The lack of viral associated miRNAs for both iciHHV-6A and 293-HHV-6A cells was a somewhat surprising result as viral miRNAs have been associated with herpesvirus latency, and have been shown to regulate the switch between latency and lytic replication (Skalsky and Cullen, 2010; Cullen, 2011; Piedade and Azevedo-Pereira, 2016). Latency-expressed miRNAs from HSV-1, EBV, and KSHV have been shown to target IE genes and thereby suppress lytic replication to maintain latency (Murphy et al., 2008; Skalsky and Cullen, 2010). Latency-specific viral miRNAs were also identified after deep sequencing of trigeminal ganglia latently infected with HSV-1; however, and similar to the results reported here, miRNAs have not been detected during VZV latency (Umbach et al., 2009). For HHV-6A and HHV-6B, virus-encoded miRNAs have been characterized in lytically infected cells (Tuddenham et al., 2012; Nukui et al., 2015). In a recent study, Prusty et al. (2018) was able to detect various viral sncRNAs in TSA-treated cells by Northern blotting, however, in the absence of detectable viral protein production or replication. Therefore, we hypothesized that viral associated transcripts, including LATs and miRNAs, would be detected upon reactivation of HHV-6A in 293-HHV-6A cells. RNA- and miRNA-seq following TPA stimulation, however, revealed that only transcripts associated with the GFP and EcoGPT regions were detected in our *in vitro* infected cells. Viral genes remained below the detection limit of RNA-seq. The increased GFP expression suggests that TPA does not stimulate transcription of HHV-6A genes, and only the foreign viral promoters in the BAC sequence are expressed in the HHV-6-GFP genome. Along with this, Liu et al. (2010) showed that TPA reverses HCMV MIE gene silencing *via* a PKC-delta-dependent mechanism in human NT2 cells. HHV-6 reactivation and associations with diseases have previously been observed in patients (Gravel et al., 2013; Endo et al., 2014; Politikos et al., 2018). Reactivation of the virus genome using various compounds *in vitro* can result in viral gene expression; however, these stimuli were not sufficient to induce the production of infectious particles in several *in vitro* infected cells (Arbuckle et al., 2010; Gravel et al., 2017; Prusty et al., 2018). Through co-cultivation studies, Arbuckle et al. (2010) demonstrated that the integrated genome in TPA- and Dexamethason-treated PBMCs from different iciHHV-6 individuals can be transmitted to Molt-3 cells. This observation may support the hypothesis that, in contrast to cells directly *ex vivo*, the HHV-6A genome in immortalized cell lines might be harder to reactivate. Taken together, the ability and efficiency of reactivation appear to be dependent on the cell type, cell line, and drug used to stimulate reactivation.

The lack of viral gene expression in our iciHHV-6A and 293-HHV-6A models suggests that a chromatin-mediated mechanism could be involved in transcriptional silencing of integrated HHV-6A. Using MNase-seq, we profiled the nucleosomal positioning across the HHV-6A genome. Results presented here demonstrate a significant decrease in MNase accessibility, which is attributed to increased nucleosomal occupancy across the majority of the viral genome. These results strongly suggest that the latent virus resides in a highly compacted state, which would exclude the necessary transcriptional machinery required for gene expression. Furthermore, ChIP assays (ChIP-seq and ChIP-qPCR) indicate that active chromatin marks (H3K4me3 and H3K27ac) were not detected above background levels in these experiments (Figure 4A), despite H3K27ac having the highest amount of total mapped reads (Table 2). Human telomeres are enriched for H3K27ac (Cubiles et al., 2018); therefore, the high number of mapped reads to HHV-6A, in the absence of H3K27ac enrichment, is likely due to non-unique reads mapping to the viral telomeric repeats. In contrast, H3K9me3 and H3K27me3 levels are relatively low in human telomeres (O’Sullivan et al., 2010; Ichikawa et al., 2015; Cubiles et al., 2018), and these repressive histone modifications show modest enrichment across the HHV-6A genome. As opposed to H3K4me3 and H3K27ac, H3K9me3 and H3K27me3 are characterized by broad low-level enrichment patterns rather than sharp, clearly enriched peaks. On viruses, peak calling is often difficult to achieve with these marks, making it difficult to distinguish enriched regions from noisy and possibly under sampled data. While the quality of repressive histone ChIP-seq experiments is affected by several experimental parameters including sonication efficiency and sequencing depth, reaching ideal experimental parameters and comparable data quality across experiments is often difficult, costly, or even impossible, resulting in low sensitivity and specificity of measurements (Jung et al., 2014). Importantly, conducting ChIP-qPCR with primers specific to several HHV-6A genomic regions confirms H3K9me3 and H3K27me3 enrichment, albeit at lower levels than cellular regions, further suggesting that these repressive histone modifications are a mechanism regulating HHV-6A latency. Interestingly, HSV or VZV infection also results in the deposition of nucleosomes carrying H3K9me3 along the viral genome (Silva et al., 2008; Kwiatkowski et al., 2009; Liang et al., 2009), and HSV-1 reactivation from latency was blocked by inhibiting the H3K9me3 histone demethylase LSD1 (Liang et al., 2009). H3K9me3 was also enriched on the latent gammaherpesvirus EBV genome, whereas relatively low levels of H3K27me3 were found (Day et al., 2007; Tempera and Lieberman, 2010). In contrast, Cliffe et al. (2009) identified H3K27me3 as the most enriched histone mark on the latent HSV-1 genome. For the HHV-6A genome, both H3K27me3 and H3K9me3 exhibit similar patterns of enrichment, with the exception of the increased H3K27me3 signal in the viral direct repeat regions. Although this is an interesting outcome, the biological relevance of this observation currently remains unknown.

## Conclusions

This study provides an unbiased integrative genomics approach toward uncovering the chromatin profile in patient-derived iciHHV-6 and experimentally infected 293-HHV-6A cell lines. Our results demonstrate that chromosomally integrated HHV-6A is completely transcriptionally silent, resides in a heterochromatic state, and is associated with H3K9me3 and H3K27me3 repressive histone modifications. Although we were unable to reactivate HHV-6A in the current study, it can be speculated that HHV-6A reactivation would alter the transcriptional state and that nucleosome occupancy would decrease across active regions of the viral genome. The lack of detectable viral associated transcripts and miRNAs in our analysis suggests that the mechanism underlying HHV-6A latency may differ significantly from those observed with other herpesviruses, e.g., HSV-1. Taken together, our data highlight how histone modifications and chromatin condensation play an important role in the control of the integrated HHV-6A genome and represent the baseline for further analysis to uncover epigenetic state during integration and reactivation.

## Data Availability

The datasets generated and analyzed for this study can be found in the NCBI short read archive under reference number SRA#GSE121987.

## Author Contributions

BK and SF designed and supervised the study and acquired funding. AS, SS, and DG performed ChIP-seq experiments. AS performed RNA-seq and MNase-seq experiments. CZ performed cell culture, qPCR, FISH, and flow cytometry experiments. AS, MM, JB, and SF performed the analysis of the sequencing data. DW, CZ, GM, AG, ID, and LF generated and provided cells. AS and CZ wrote the manuscript. AS, CZ, BK, SF, LF, and DW revised the manuscript.

### Conflict of Interest Statement

The authors declare that the research was conducted in the absence of any commercial or financial relationships that could be construed as a potential conflict of interest.

## References

[ref1] Ablashi D. Agut H. Alvarez-Lafuente R. Clark D. A. Dewhurst S. DiLuca D.. (2014). Classification of HHV-6A and HHV-6B as distinct viruses. Arch. Virol. 159, 863–870. 10.1007/s00705-013-1902-5, PMID: 24193951 PMC4750402

[ref2] Adams M. J. Carstens E. B. (2012). Ratification vote on taxonomic proposals to the International Committee on Taxonomy of Viruses. Arch. Virol. 157, 1411–1422. 10.1007/s00705-012-1299-6, PMID: 22481600 PMC7086667

[ref3] Anders S. Pyl P. T. Huber W. (2015). HTSeq—a Python framework to work with high-throughput sequencing data. Bioinformatics 31, 166–169. 10.1093/bioinformatics/btu638, PMID: 25260700 PMC4287950

[ref4] Arbuckle J. H. Medveczky M. M. Luka J. Hadley S. H. Luegmayr A. Ablashi D. (2010). The latent human herpesvirus-6A genome specifically integrates in telomeres of human chromosomes in vivo and in vitro. Proc. Natl. Acad. Sci. USA 107, 5563–5568. 10.1073/pnas.0913586107 20212114 PMC2851814

[ref5] Arbuckle J. H. Pantry S. N. Medveczky M. M. Prichett J. Loomis K. S. Ablashi D.. (2013). Mapping the telomere integrated genome of human herpesvirus 6A and 6B. Virology 442, 3–11. 10.1016/j.virol.2013.03.030, PMID: 23648233 PMC3696530

[ref6] Berger S. L. (2007). The complex language of chromatin regulation during transcription. Nature 447, 407–412. 10.1038/nature05915, PMID: 17522673

[ref7] Bloom D. C. Giordani N. V. Kwiatkowski D. L. (2010). Epigenetic regulation of latent HSV-1 gene expression. Biochim. Biophys. Acta 1799, 246–256. 10.1016/j.bbagrm.2009.12.001, PMID: 20045093 PMC2838971

[ref8] Buenrostro J. D. Giresi P. G. Zaba L. C. Chang H. Y. Greenleaf W. J. (2013). Transposition of native chromatin for fast and sensitive epigenomic profiling of open chromatin, DNA-binding proteins and nucleosome position. Nat. Methods 10, 1213–1218. 10.1038/nmeth.2688, PMID: 24097267 PMC3959825

[ref9] Caselli E. Di Luca D. (2007). Molecular biology and clinical associations of Roseoloviruses human herpesvirus 6 and human herpesvirus 7. New Microbiol. 30, 173–187. PMID: https://www.ncbi.nlm.nih.gov/pubmed/17802896 17802896

[ref10] Chen K. Xi Y. Pan X. Li Z. Kaestner K. Tyler J.. (2013). DANPOS: dynamic analysis of nucleosome position and occupancy by sequencing. Genome Res. 23, 341–351. 10.1101/gr.142067.112, PMID: 23193179 PMC3561875

[ref11] Cheng S. Caviness K. Buehler J. Smithey M. Nikolich-Žugich J. Goodrum F. (2017). Transcriptome-wide characterization of human cytomegalovirus in natural infection and experimental latency. Proc. Natl. Acad. Sci. USA 114, E10586–E10595. 10.1073/pnas.1710522114, PMID: 29158406 PMC5724264

[ref12] Cliffe A. R. Garber D. A. Knipe D. M. (2009). Transcription of the herpes simplex virus latency-associated transcript promotes the formation of facultative heterochromatin on lytic promoters. J. Virol. 83, 8182–8190. 10.1128/JVI.00712-09, PMID: 19515781 PMC2715743

[ref13] Collins-McMillen D. Goodrum F. D. (2017). The loss of binary: pushing the herpesvirus latency paradigm. Curr. Clin. Microbiol. Rep. 4, 124–131. 10.1007/s40588-017-0072-8, PMID: 29250481 PMC5726573

[ref14] Cubiles M. D. Barroso S. Vaquero-Sedas M. I. Enguix A. Aguilera A. Vega-Palas M. A. (2018). Epigenetic features of human telomeres. Nucleic Acids Res. 46, 2347–2355. 10.1093/nar/gky006, PMID: 29361030 PMC5861411

[ref15] Cui K. Zhao K. (2012). “Genome-wide approaches to determining nucleosome occupancy in metazoans using MNase-Seq” in Chromatin remodeling. ed. Morse R. (Totowa, NJ: Humana Press), 413–419. 10.1007/978-1-61779-477-3_24 PMC3541821 22183607

[ref16] Cullen B. R. (2011). Herpesvirus microRNAs: phenotypes and functions. Curr. Opin. Virol. 1, 211–215. 10.1016/j.coviro.2011.04.003, PMID: 21927637 PMC3171754

[ref17] Day L. Chau C. M. Nebozhyn M. Rennekamp A. J. Showe M. Lieberman P. M. (2007). Chromatin profiling of Epstein-Barr virus latency control region. J. Virol. 81, 6389–6401. 10.1128/JVI.02172-06, PMID: 17409162 PMC1900095

[ref18] De Bolle L. Naesens L. De Clercq E. (2005). Update on human herpesvirus 6 biology, clinical features, and therapy. Clin. Microbiol. Rev. 18, 217–245. 10.1128/CMR.18.1.217-245.2005, PMID: 15653828 PMC544175

[ref20] Depledge D. P. Ouwendijk W. J. Sadaoka T. Braspenning S. E. Mori Y. Cohrs R. J.. (2018). A spliced latency-associated VZV transcript maps antisense to the viral transactivator gene 61. Nat. Commun. 9:1167. 10.1038/s41467-018-03569-2, PMID: 29563516 PMC5862956

[ref21] Deshmane S. L. Fraser N. W. (1989). During latency, herpes simplex virus type 1 DNA is associated with nucleosomes in a chromatin structure. J. Virol. 63, 943–947. PMID: 2536115 10.1128/jvi.63.2.943-947.1989 PMC247770

[ref22] du Chéné I. Basyuk E. Lin Y. L. Triboulet R. Knezevich A. Chable-Bessia C.. (2007). Suv39H1 and HP1γ are responsible for chromatin-mediated HIV-1 transcriptional silencing and post-integration latency. EMBO J. 26, 424–435. 10.1038/sj.emboj.7601517, PMID: 17245432 PMC1783455

[ref23] Dyson P. J. Farrell P. J. (1985). Chromatin structure of Epstein-Barr virus. J. Gen. Virol. 66, 1931–1940. 10.1099/0022-1317-66-9-1931, PMID: 2993484

[ref24] Endo A. Watanabe K. Ohye T. Suzuki K. Matsubara T. Shimizu N.. (2014). Molecular and virological evidence of viral activation from chromosomally integrated human herpesvirus 6A in a patient with X-linked severe combined immunodeficiency. Clin. Infect. Dis. 59, 545–548. 10.1093/cid/ciu323, PMID: 24803376

[ref25] Friedman J. Cho W. K. Chu C. K. Keedy K. S. Archin N. M. Margolis D. M.. (2011). Epigenetic silencing of HIV-1 by the histone H3 lysine 27 methyltransferase enhancer of Zeste 2. J. Virol. 85, 9078–9089. 10.1128/JVI.00836-11, PMID: 21715480 PMC3165831

[ref26] Goodrum F. (2016). Human cytomegalovirus latency: approaching the Gordian Knot. Annu. Rev. Virol. 3, 333–357. 10.1146/annurev-virology-110615-042422, PMID: 27501258 PMC5514425

[ref27] Gravel A. Dubuc I. Morissette G. Sedlak R. H. Jerome K. R. Flamand L. (2015). Inherited chromosomally integrated human herpesvirus 6 as a predisposing risk factor for the development of angina pectoris. Proc. Natl. Acad. Sci. USA 112, 8058–8063. 10.1073/pnas.1502741112 26080419 PMC4491735

[ref28] Gravel A. Dubuc I. Wallaschek N. Gilbert-Girard S. Collin V. Hall-Sedlak R. (2017). Cell culture systems to study Human herpesvirus 6A/B chromosomal integration. J. Virol. 91, e00437–e00417. 10.1128/JVI.00437-17 28468878 PMC5487542

[ref29] Gravel A. Hall C. B. Flamand L. (2013). Sequence analysis of transplacentally acquired human herpesvirus 6 DNA is consistent with transmission of a chromosomally integrated reactivated virus. J. Infect. Dis. 207, 1585–1589. 10.1093/infdis/jit060, PMID: 23408849

[ref30] Grinde B. (2013). Herpesviruses: latency and reactivation - viral strategies and host response. J. Oral Microbiol. 5:22766. 10.3402/jom.v5i0.22766, PMID: 24167660 PMC3809354

[ref31] Hill J. A. Magaret A. S. Hall-Sedlak R. Mikhaylova A. Huang M. L. Sandmaier B. M.. (2017). Outcomes of hematopoietic cell transplantation using donors or recipients with inherited chromosomally integrated HHV-6. Blood 130, 1062–1069. 10.1182/blood-2017-03-775759, PMID: 28596425 PMC5570681

[ref32] Hill J. A. Zerr D. M. (2014). Roseoloviruses in transplant recipients: clinical consequences and prospects for treatment and prevention trials. Curr. Opin. Virol. 9, 53–60. 10.1016/j.coviro.2014.09.006 25285614 PMC4570620

[ref33] Ichikawa Y. Nishimura Y. Kurumizaka H. Shimizu M. (2015). Nucleosome organization and chromatin dynamics in telomeres. Biomol. Concepts 6, 67–75. 10.1515/bmc-2014-0035, PMID: 25720088

[ref34] Jiang C. Pugh B. F. (2009). Nucleosome positioning and gene regulation: advances through genomics. Nat. Rev. Genet. 10, 161–172. 10.1038/nrg2522, PMID: 19204718 PMC4860946

[ref35] Jordan A. Bisgrove D. Verdin E. (2003). HIV reproducibly establishes a latent infection after acute infection of T cells in vitro. EMBO J. 22, 1868–1877. 10.1093/emboj/cdg188, PMID: 12682019 PMC154479

[ref36] Jung Y. L. Luquette L. J. Ho J. W. Ferrari F. Tolstorukov M. Minoda A.. (2014). Impact of sequencing depth in ChIP-seq experiments. Nucleic Acids Res. 42:e74. 10.1093/nar/gku178, PMID: 24598259 PMC4027199

[ref37] Kaufer B. B. (2013). “Detection of integrated herpesvirus genomes by fluorescence in situ hybridization (FISH)” in Virus-host interactions. eds. Bailer S. Lieber D. (Totowa, NJ: Humana Press), 141–152. 10.1007/978-1-62703-601-6_10 23996255

[ref38] Kaufer B. B. Flamand L. (2014). Chromosomally integrated HHV-6: impact on virus, cell and organismal biology. Curr. Opin. Virol. 9, 111–118. 10.1016/j.coviro.2014.09.010, PMID: 25462442

[ref39] Kaufer B. B. Jarosinski K. W. Osterrieder N. (2011). Herpesvirus telomeric repeats facilitate genomic integration into host telomeres and mobilization of viral DNA during reactivation. J. Exp. Med. 208, 605–615. 10.1084/jem.20101402, PMID: 21383055 PMC3058580

[ref40] Kinchington P. R. St Leger A. J. Guedon J. M. G. Hendricks R. L. (2012). Herpes simplex virus and varicella zoster virus, the house guests who never leave. Herpesviridae 3:5. 10.1186/2042-4280-3-5, PMID: 22691604 PMC3541251

[ref41] Knipe D. M. Cliffe A. (2008). Chromatin control of herpes simplex virus lytic and latent infection. Nat. Rev. Microbiol. 6, 211–221. 10.1038/nrmicro1794, PMID: 18264117

[ref42] Kondo K. Sashihara J. Shimada K. Takemoto M. Amo K. Miyagawa H.. (2003). Recognition of a novel stage of betaherpesvirus latency in human herpesvirus 6. J. Virol. 77, 2258–2264. 10.1128/JVI.77.3.2258-2264.2003, PMID: 12525662 PMC140895

[ref43] Kondo K. Shimada K. Sashihara J. Tanaka-Taya K. Yamanishi K. (2002). Identification of human herpesvirus 6 latency-associated transcripts. J. Virol. 76, 4145–4151. 10.1128/JVI.76.8.4145-4151.2002, PMID: 11907257 PMC136062

[ref44] Kubat N. J. Tran R. K. McAnany P. Bloom D. C. (2004). Specific histone tail modification and not DNA methylation is a determinant of herpes simplex virus type 1 latent gene expression. J. Virol. 78, 1139–1149. 10.1128/JVI.78.3.1139-1149.2004, PMID: 14722269 PMC321404

[ref45] Kwiatkowski D. L. Thompson H. W. Bloom D. C. (2009). The polycomb group protein Bmi1 binds to the herpes simplex virus 1 latent genome and maintains repressive histone marks during latency. J. Virol. 83, 8173–8181. 10.1128/JVI.00686-09, PMID: 19515780 PMC2715759

[ref46] Langmead B. Salzberg S. L. (2012). Fast gapped-read alignment with Bowtie 2. Nat. Methods 9, 357–359. 10.1038/nmeth.1923, PMID: 22388286 PMC3322381

[ref47] Li B. Carey M. Workman J. L. (2007). The role of chromatin during transcription. Cell 128, 707–719. 10.1016/j.cell.2007.01.015, PMID: 17320508

[ref48] Li H. Handsaker B. Wysoker A. Fennell T. Ruan J. Homer N.. (2009). The sequence alignment/map format and SAMtools. Bioinformatics 25, 2078–2079. 10.1093/bioinformatics/btp352, PMID: 19505943 PMC2723002

[ref49] Liang Y. Vogel J. L. Narayanan A. Peng H. Kristie T. M. (2009). Inhibition of the histone demethylase LSD1 blocks alpha-herpesvirus lytic replication and reactivation from latency. Nat. Med. 15, 1312–1317. 10.1038/nm.2051, PMID: 19855399 PMC2783573

[ref50] Liu X. Yuan J. Wu A. W. McGonagill P. W. Galle C. S. Meier J. L. (2010). Phorbol ester-induced human cytomegalovirus major immediate-early (MIE) enhancer activation through PKC-delta, CREB, and NF-kappaB desilences MIE gene expression in quiescently infected human pluripotent NTera2 cells. J. Virol. 84, 8495–8508. 10.1128/JVI.00416-10, PMID: 20504934 PMC2919020

[ref51] Love M. I. Huber W. Anders S. (2014). Moderated estimation of fold change and dispersion for RNA-seq data with DESeq2. Genome Biol. 15:550. 10.1186/s13059-014-0550-8, PMID: 25516281 PMC4302049

[ref52] Lusso P. Malnati M. De Maria A. Balotta C. Derocco S. E. Markham P. D.. (1991). Productive infection of CD4+ and CD8+ mature human T cell populations and clones by human herpesvirus 6. Transcriptional down-regulation of CD3. J. Immunol. 147, 685–691. PMID: . 1677024

[ref54] Maehara K. Ohkawa Y. (2016). Exploration of nucleosome positioning patterns in transcription factor function. Sci. Rep. 6:19620. 10.1038/srep19620, PMID: 26790608 PMC4726364

[ref55] Mieczkowski J. Cook A. Bowman S. K. Mueller B. Alver B. H. Kundu S.. (2016). MNase titration reveals differences between nucleosome occupancy and chromatin accessibility. Nat. Commun. 7:11485. 10.1038/ncomms11485, PMID: 27151365 PMC4859066

[ref56] Moquin S. A. Thomas S. Whalen S. Warburton A. Fernandez S. G. McBride A. A. (2018). The Epstein-Barr virus episome maneuvers between nuclear chromatin compartments during reactivation. J. Virol. 92, e01413–e01417. 10.1128/JVI.01413-17 29142137 PMC5774889

[ref57] Moreau M. E. Bawolak M. T. Morissette G. Adam A. Marceau F. (2007). Role of nuclear factor-κB and protein kinase C signaling in the expression of the kinin B1 receptor in human vascular smooth muscle cells. Mol. Pharmacol. 71, 949–956. 10.1124/mol.106.030684, PMID: 17178924

[ref58] Mueller B. Mieczkowski J. Kundu S. Wang P. Sadreyev R. Tolstorukov M. Y.. (2017). Widespread changes in nucleosome accessibility without changes in nucleosome occupancy during a rapid transcriptional induction. Genes Dev. 31, 451–462. 10.1101/gad.293118.116, PMID: 28356342 PMC5393060

[ref59] Murphy E. Vaníček J. Robins H. Shenk T. Levine A. J. (2008). Suppression of immediate-early viral gene expression by herpesvirus-coded microRNAs: implications for latency. Proc. Natl. Acad. Sci. USA 105, 5453–5458. 10.1073/pnas.0711910105 18378902 PMC2291141

[ref60] Nicol J. W. Helt G. A. Blanchard, Jr. S. G. Raja A. Loraine A. E. (2009). The Integrated Genome Browser: free software for distribution and exploration of genome-scale datasets. Bioinformatics 25, 2730–2731. 10.1093/bioinformatics/btp472, PMID: 19654113 PMC2759552

[ref61] Nukui M. Mori Y. Murphy E. A. (2015). A human herpesvirus 6A-encoded microRNA: role in viral lytic replication. J. Virol. 89, 2615–2627. 10.1128/JVI.02007-14, PMID: 25520507 PMC4325741

[ref62] O’Geen H. Frietze S. Farnham P. J. (2010). “Using ChIP-seq technology to identify targets of zinc finger transcription factors” in Engineered zinc finger proteins: methods and protocols. eds. Mackay J. Segal D. (Totowa, NJ: Humana Press), 437–455. 10.1007/978-1-60761-753-2_27 PMC4151297 20680851

[ref63] O’Sullivan R. J. Kubicek S. Schreiber S. L. Karlseder J. (2010). Reduced histone biosynthesis and chromatin changes arising from a damage signal at telomeres. Nat. Struct. Mol. Biol. 17, 1218–1225. 10.1038/nsmb.1897, PMID: 20890289 PMC2951278

[ref64] Pajoro A. Muiño J. M. Angenent G. C. Kaufmann K. (2018). “Profiling nucleosome occupancy by MNase-seq: experimental protocol and computational analysis” in Plant chromatin dynamics. Methods in Molecular Biology. eds. Bemer M. Baroux C., Vol. 1675 (New York, NY: Humana Press). 10.1007/978-1-4939-7318-7_11 29052192

[ref65] Pellett P. E. Ablashi D. V. Ambros P. F. Agut H. Caserta M. T. Descamps V.. (2012). Chromosomally integrated human herpesvirus 6: questions and answers. Rev. Med. Virol. 22, 144–155. 10.1002/rmv.715, PMID: 22052666 PMC3498727

[ref66] Piedade D. Azevedo-Pereira J. M. (2016). The role of microRNAs in the pathogenesis of herpesvirus infection. Viruses 8:156. 10.3390/v8060156, PMID: 27271654 PMC4926176

[ref67] Politikos I. McMasters M. Bryke C. Avigan D. Boussiotis V. A. (2018). Possible reactivation of chromosomally integrated human herpesvirus 6 after treatment with histone deacetylase inhibitor. Blood Adv. 2, 1367–1370. 10.1182/bloodadvances.2018015982, PMID: 29898877 PMC6020801

[ref68] Prusty B. K. Gulve N. Chowdhury S. R. Schuster M. Strempel S. Descamps V.. (2018). HHV-6 encoded small non-coding RNAs define an intermediate and early stage in viral reactivation. NPJ Genom. Med. 3:25. 10.1038/s41525-018-0064-5, PMID: 30210807 PMC6125432

[ref69] Rahman R. Gautam A. Bethune J. Sattar A. Fiosins M. Magruder D. S.. (2018). Oasis 2: improved online analysis of small RNA-seq data. BMC Bioinform. 19:54. 10.1186/s12859-018-2047-z, PMID: 29444641 PMC5813365

[ref70] Ramírez F. Ryan D. P. Grüning B. Bhardwaj V. Kilpert F. Richter A. S.. (2016). deepTools2: a next generation web server for deep-sequencing data analysis. Nucleic Acids Res. 44, W160–W165. 10.1093/nar/gkw257, PMID: 27079975 PMC4987876

[ref71] Readhead B. Haure-Mirande J. V. Funk C. C. Richards M. A. Shannon P. Haroutunian V.. (2018). Multiscale analysis of independent alzheimer’s cohorts finds disruption of molecular, genetic, and clinical networks by human herpesvirus. Neuron 99, 64–82.e7. 10.1016/j.neuron.2018.05.023, PMID: 29937276 PMC6551233

[ref72] Rens W. Fu B. O’Brien P. C. Ferguson-Smith M. (2006). Cross-species chromosome painting. Nat. Protoc. 1, 783–790. 10.1038/nprot.2006.91, PMID: 17406308

[ref73] Rotola A. Ravaioli T. Gonelli A. Dewhurst S. Cassai E. Di Luca D. (1998). U94 of human herpesvirus 6 is expressed in latently infected peripheral blood mononuclear cells and blocks viral gene expression in transformed lymphocytes in culture. Proc. Natl. Acad. Sci. USA 95, 13911–13916. 10.1073/pnas.95.23.13911 9811900 PMC24961

[ref74] Salahuddin S. Z. Ablashi D. V. Markham P. D. Josephs S. F. Sturzenegger S. Kaplan M. (1986). Isolation of a new virus, HBLV, in patients with lymphoproliferative disorders. Science 234, 596–601. 10.1126/science.2876520 2876520

[ref75] Shaw J. E. (1985). The circular intracellular form of Epstein-Barr virus DNA is amplified by the virus-associated DNA polymerase. J. Virol. 53, 1012–1015. PMID: 2983082 10.1128/jvi.53.3.1012-1015.1985 PMC254746

[ref76] Shnayder M. Nachshon A. Krishna B. Poole E. Boshkov A. Binyamin A. (2018). Defining the transcriptional landscape during cytomegalovirus latency with single-cell RNA sequencing. MBio 9, e00013–e00018. 10.1128/mBio.00013-18 29535194 PMC5850328

[ref77] Silva L. Cliffe A. Chang L. Knipe D. M. (2008). Role for A-type lamins in herpesviral DNA targeting and heterochromatin modulation. PLoS Pathog. 4:e1000071. 10.1371/journal.ppat.1000071, PMID: 18497856 PMC2374905

[ref78] Skalsky R. L. Cullen B. R. (2010). Viruses, microRNAs, and host interactions. Annu. Rev. Microbiol. 64, 123–141. 10.1146/annurev.micro.112408.134243, PMID: 20477536 PMC3621958

[ref79] Strenger V. Caselli E. Lautenschlager I. Schwinger W. Aberle S. W. Loginov R.. (2014). Detection of HHV-6-specific mRNA and antigens in PBMCs of individuals with chromosomally integrated HHV-6 (ciHHV-6). Clin. Microbiol. Infect. 20, 1027–1032. 10.1111/1469-0691.12639, PMID: 24698304

[ref80] Takahashi K. Sonoda S. Higashi K. Kondo T. Takahashi H. Takahashi M.. (1989). Predominant CD4 T-lymphocyte tropism of human herpesvirus 6-related virus. J. Virol. 63, 3161–3163. PMID: . 2542623 10.1128/jvi.63.7.3161-3163.1989 PMC250875

[ref81] Tang H. Kawabata A. Yoshida M. Oyaizu H. Maeki T. Yamanishi K.. (2010). Human herpesvirus 6 encoded glycoprotein Q1 gene is essential for virus growth. Virology 407, 360–367. 10.1016/j.virol.2010.08.018, PMID: 20863544

[ref82] Tempera I. Lieberman P. M. (2010). Chromatin organization of gammaherpesvirus latent genomes. Biochim. Biophys. Acta 1799, 236–245. 10.1016/j.bbagrm.2009.10.004, PMID: 19853673 PMC2839031

[ref83] Trapnell C. Pachter L. Salzberg S. L. (2009). TopHat: discovering splice junctions with RNA-Seq. Bioinformatics 25, 1105–1111. 10.1093/bioinformatics/btp120, PMID: 19289445 PMC2672628

[ref84] Tuddenham L. Jung J. S. Chane-Woon-Ming B. Dölken L. Pfeffer S. (2012). Small RNA deep sequencing identifies microRNAs and other small noncoding RNAs from human herpesvirus 6B. J. Virol. 86, 1638–1649. 10.1128/JVI.05911-11, PMID: 22114334 PMC3264354

[ref85] Umbach J. L. Nagel M. A. Cohrs R. J. Gilden D. H. Cullen B. R. (2009). Analysis of human alphaherpesvirus microRNA expression in latently infected human trigeminal ganglia. J. Virol. 83, 10677–10683. 10.1128/JVI.01185-09, PMID: 19656888 PMC2753103

[ref86] Vinnard C. Barton T. Jerud E. Blumberg E. (2009). A report of human herpesvirus 6-associated encephalitis in a solid organ transplant recipient and a review of previously published cases. Liver Transpl. 15, 1242–1246. 10.1002/lt.21816, PMID: 19790143

[ref87] Wallaschek N. Sanyal A. Pirzer F. Gravel A. Mori Y. Flamand L.. (2016). The telomeric repeats of human herpesvirus 6A (HHV-6A) are required for efficient virus integration. PLoS Pathog. 12:e1005666. 10.1371/journal.ppat.1005666, PMID: 27244446 PMC4887096

[ref88] Wang Q. Y. Zhou C. Johnson K. E. Colgrove R. C. Coen D. M. Knipe D. M. (2005). Herpesviral latency-associated transcript gene promotes assembly of heterochromatin on viral lytic-gene promoters in latent infection. Proc. Natl. Acad. Sci. USA 102, 16055–16059. 10.1073/pnas.0505850102 16247011 PMC1266038

[ref89] Wight D. J. Wallaschek N. Sanyal A. Weller S. K. Flamand L. Kaufer B. B. (2018). Viral Proteins U41 and U70 of human herpesvirus 6A are dispensable for telomere integration. Viruses 10:656. 10.3390/v10110656, PMID: 30469324 PMC6267051

[ref90] Winestone L. E. Punn R. Tamaresis J. S. Buckingham J. Pinsky B. A. Waggoner J. J.. (2018). High human herpesvirus 6 viral load in pediatric allogeneic hematopoietic stem cell transplant patients is associated with detection in end organs and high mortality. Pediatr. Transplant. 22:e13084. 10.1111/petr.13084, PMID: 29181879 PMC5820136

[ref91] Yamanishi K. Okuno T. Shiraki K. Takahashi M. Kondo T. Asano Y.. (1988). Identification of human herpesvirus-6 as a causal agent for exanthem subitum. Lancet 1, 1065–1067. 10.1016/S0140-6736(88)91893-4, PMID: 2896909

[ref92] Zerr D. M. Meier A. S. Selke S. S. Frenkel L. M. Huang M.-L. Wald A.. (2005). A population-based study of primary human herpesvirus 6 infection. N. Engl. J. Med. 352, 768–776. 10.1056/NEJMoa042207, PMID: 15728809

[ref93] Zhang Y. Liu T. Meyer C. A. Eeckhoute J. Johnson D. S. Bernstein B. E.. (2008). Model-based analysis of ChIP-Seq (MACS). Genome Biol. 9:R137. 10.1186/gb-2008-9-9-r137, PMID: 18798982 PMC2592715

