# Supplementary material for: Chromatin Profiles of Chromosomally Integrated Human Herpesvirus-6A
Source: Front Microbiol. 2019 Jun 26;10:1408. doi: 10.3389/fmicb.2019.01408 (PMC6606781; doi:10.3389/fmicb.2019.01408)
Supplement: Supplementary file 1 [file Data_Sheet_1.pdf]

Supplementary Material

Supplementary Figures

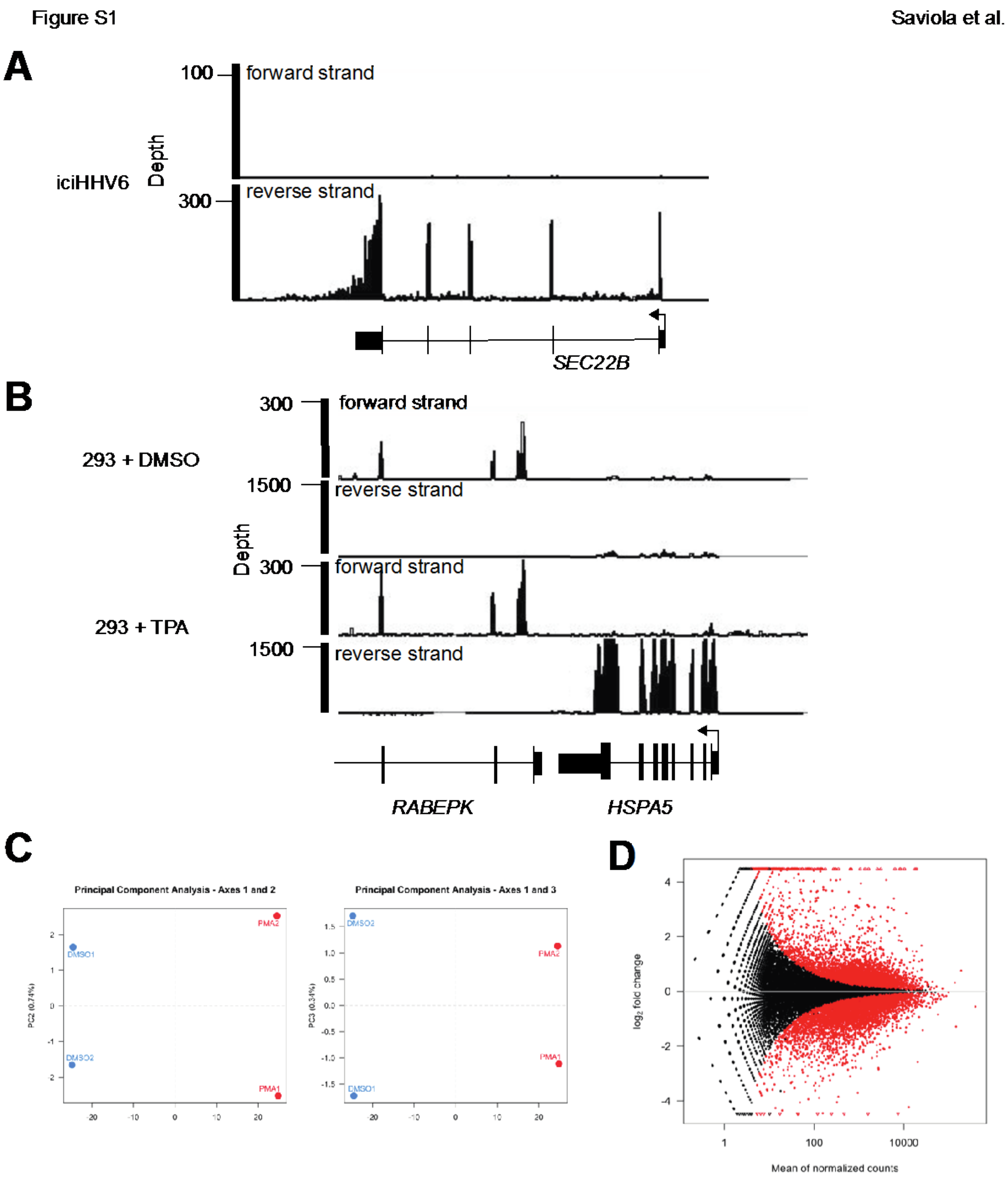

Supplementary Figure S1 | RNA-seq results for iciHHV-6A cells and 293-HHV-6A cells. **(A)** Snapshot of forward and reverse strands of the human *SEC22B* gene in Integrated Genome Browser from iciHHV-6A RNA-seq data. **(B)** Snapshot of the human *HSPA5* gene locus showing increased

expression in the TPA- vs DMSO-treated 293-HHV-6A cells. **(C)** First two components of a Principal Component Analysis, with percentages of variance associated with each axis. **(D)** DESeq2 differential expression MA-plot of TPA- vs DMSO-treated 293-HHV-6A cells. Red dots represent significantly differentially expressed features. Triangles correspond to features having a too high of log2 fold change to be displayed on the plot.

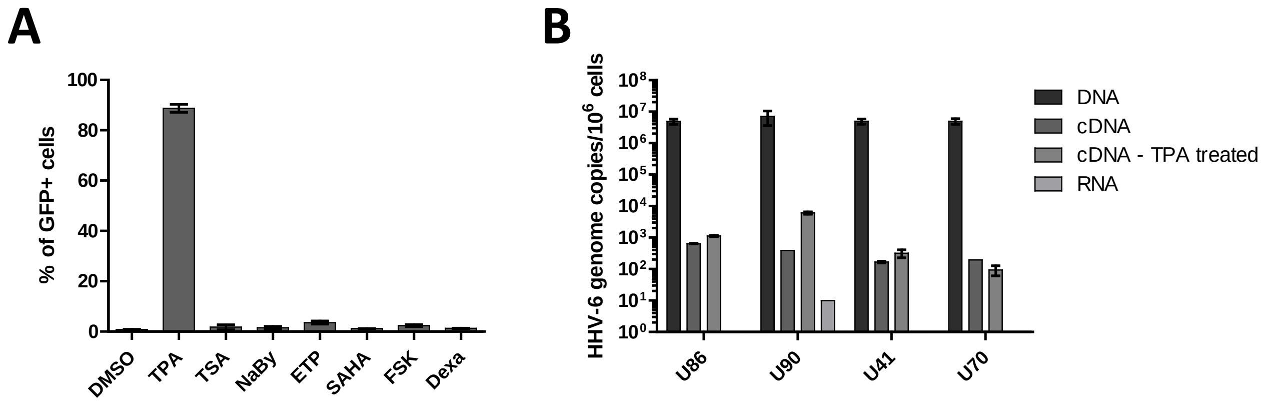

Supplementary Figure S2 | TPA stimulation induces GFP expression in latently infected 293 T cells. **(A)** 293-HHV-6A cells were treated with indicated reagents for 24 h. GFP expression was measured by flow cytometry. Mean of the % of GFP+ cells from three independent experiments are shown. **(B)** 293-HHV-6A cells were treated with TPA or DMSO for 6 days. HHV-6A gene expression was detected by RT-qPCR. Copy numbers per million cells are shown as means of two independent experiments.

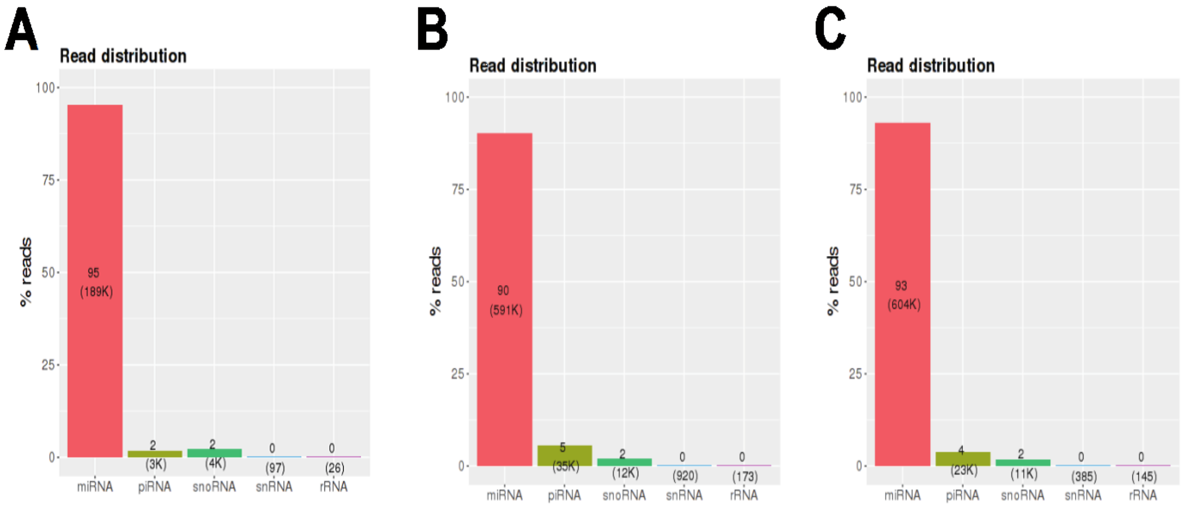

Supplementary Figure S3 | miRNA analysis showing the percentage of uniquely aligned reads per small RNA species for (**A)** iciHHV-6A and 293-HHV-6A cells following (**B)** DMSO or **(C)** TPA treatment. RNA species considered are miRNAs, piRNAs, snoRNAs, snRNAs, and rRNAs. For all conditions ≥ 90% of the useable reads were classified as miRNAs.
